# Anti-neutrophil cytoplasmic antibody associated vasculitis of the brain and oral cavity: a case report

**DOI:** 10.1093/omcr/omad100

**Published:** 2023-09-25

**Authors:** D Bontempo, A M Frydrych, O Kujan, D Gebauer, M Fallon, P K Panegyres

**Affiliations:** School of Medicine, The University of Western Australia, Nedlands, Australia; UWA Dental School, The University of Western Australia, Nedlands, Australia; UWA Dental School, The University of Western Australia, Nedlands, Australia; Department of Oral and Maxillofacial Surgery, Royal Perth Hospital, Perth, Australia; Department of Human Sciences, Oral Health and Equity, University of Western Australia, Perth, Australia; Perth Radiological Clinic, Subiaco, Australia; School of Medicine, The University of Western Australia, Nedlands, Australia; Neurodegenerative Disorders Research Pty Ltd, West Perth, Australia

## Abstract

We report a patient with a novel presentation of anti-neutrophil cytoplasmic antibody positive (ANCA+) vasculitis of the brain and oral mucosa. ANCA+ vasculitis of the brain is usually associated with pachymeningitis and hypophysitis, and there are no cases reported with simultaneous brain and oral mucosal involvement. A 35-year-old African Zambian man presented with headache and bleeding swollen gingiva. He was myeloperoxidase (MPO) antibody positive with cytoplasmic staining. His MRI showed stable small callosal, periventricular and subcortical white matter non-enhancing lesions, without change over 15 months—compatible with vasculitis. His gingival biopsy was consistent with vasculitis. His headache and oral lesions responded to oral corticosteroids and intravenous immunoglobulin which have induced clinical remission. Our patient expands the clinical syndrome of ANCA+ MPO+ C-type vasculitis of the central nervous system with headaches complicating cerebral vasculitis and oral mucosal involvement.

## INTRODUCTION

Anti-neutrophil cytoplasmic antibody positive (ANCA+) involvement of the central nervous system (CNS) is usually characterized by hypertrophic pachymeningitis, strokes, hypophysitis, posterior reversible encephalopathy syndrome, mass lesions or myelopathy [1].

## CASE REPORT

A 35-year-old African Zambian man was referred for assessment of recurrent left-sided headaches of two-years’ duration without other constitutional symptoms such as anorexia, fatigue, night sweats or malaise.

**Figure 1:**
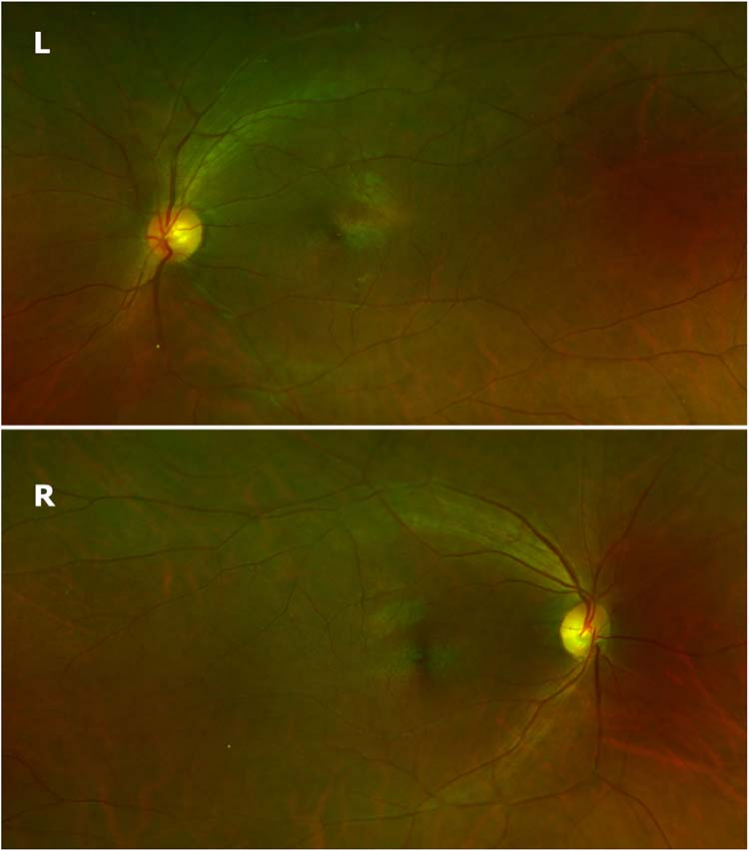
Fundal photographs showing asymmetrical optic pallor in June 2021, prior to the initiation of steroids.

The examination revealed visual acuity of J2 in both eyes. Pallor of optic discs was more marked on the right than the left (see Fig. 1). The pupils were 3 mm on both sides with normal direct and consensual light responses. Visual fields were full. His eye movements were normal. The remainder of the neurological examination and general physical examination were normal.

**Figure 2:**
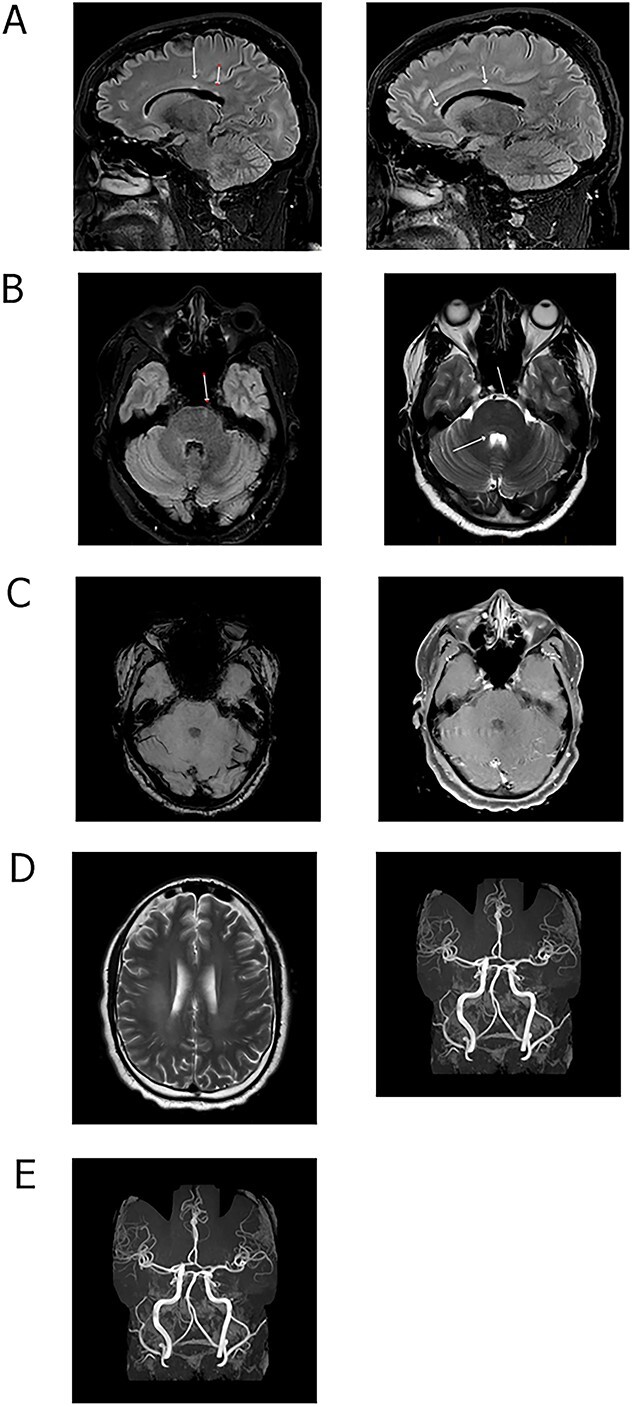
MRI scans of patient with ANCA+ MPO+ vasculitis of the brain and oral cavity. (**A**) Sagittal FLAIR images. (**B**) Axial FLAIR and T2-weighted images. (**C**) Axial SWI and contrast enhanced T1-weighted images. (**D**) Axial FLAIR and T2-weighted images; and (**E**) MR angiogram image at presentation.

Five weeks after his initial presentation in April 2021, the left-sided headache persisted and he complained of exhaustion. The MRI showed small callosal, periventricular and subcortical white matter lesions in the supratentorial compartment with subtle poorly defined hyperintensities in the centrum semiovale on the right side. Subtle T2 hyperintensities abutting the cerebral aqueduct and the fourth ventricular floor were noted. There was asymmetrical T2-signal hyperintensity of the right optic nerve. No pathological cerebral parenchymal or leptomeningeal enhancement were identified (Fig. 2). The MRI did not disclose vessel wall enhancement, vascular irregularity or haemorrhages. His MPO antibody was positive at 32.4 units (ref range < 3.5 U ml^−1^) with cytoplasmic staining: the anti-PR3 was negative—these laboratory findings were confirmed three times.

His erythrocyte sedimentation rate and C-reactive protein were normal. His renal and liver function tests, thyroid antibodies, thyroid function tests, antinuclear and anti-extractable nuclear antigen (anti-ENAs), antibodies, C3/C4, anticardiolipin and antiphospholipid antibodies, anti-Beta 2 glycoprotein I, IgG, anti-glomerular basement membrane antibodies were normal or negative; angiotensin converting enzyme was negative and B12 folate was normal. HIV and Syphilis serology were negative. Anti-aquaporin 4 and anti-MOG antibodies were negative; he was human leukocyte antigen (HLA) type DRB1*13. The cerebrospinal fluid (CSF) showed a protein of 0.34 g l^−1^ (0.15–0.45) with CSF glucose 3.4 mmol l^−1^ (2.7–4.4), and concurrent blood glucose 5 mmol l^−1^. CSF white blood count was 3 × 10*6 l^−1^, red blood cell count was 8 × 10*6 l^−1^ with negative Gram stain and culture, with oligoclonal bands (OGCBs). Cytology was negative for malignancy but did show an increase in lymphocytes, with isolated monocytes. Spinal MR was normal.

**Figure 3:**
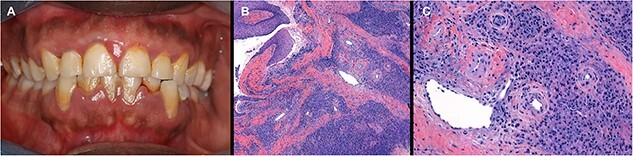
(**A**) Gingival swelling and bleeding. (**B**) H&E ×100; and (**C**) H&E × 400 showing mid and deep gingival biopsy with extensive inflammatory infiltrate of histocytes and neutrophils. Several small blood vessels show thickened walls due to fibrinoid necrosis.

Four weeks after his presentation, and armed with the findings of CSF analysis and ANCA results, he was commenced on prednisolone 50 mg per day at a reducing dose. As the dose of prednisolone was lowered to 10 mg per day—about two months after presentation—his headaches had resolved but he reported that his gingiva were swollen and bleeding. He did not disclose this on first presentation as he did not believe it was relevant. As the prednisolone was reduced further, he had an increase in headaches and increase in gingival swelling and bleeding (Fig. 3A). He never had the clinical syndrome of optic neuritis –optic nerve pallor is recognized in vasculitis through the mechanism of arteritic ischaemic optic neuropathy.

The possibility of vasculitis of the brain and oral cavity were considered. He then reported spontaneous gingival bleeding and swelling, roughly over the two years of duration of his headaches. His partner had noticed spontaneous gingival bleeding during conversation. He had been aware of spontaneous gingival bleeding while eating. He was unable to brush his teeth because of gingival bleeding. He would awaken with the taste of blood.

A gingival biopsy, while on prednisolone, showed extensive inflammatory infiltrate of histocytes and neutrophils, and thickened small blood vessel walls due to fibrinoid necrosis with immunofluorescence reactive IgA and IgM in the dermis, with C3 in the dermis blood vessels consistent with vasculitis (Fig. 3B and C).

After the biopsy he was commenced on intravenous immunoglobulin—10% with 0.4 g kg^−1^ per day for five days, then 0.4 g kg^−1^ per day as monthly maintenance—as the patient declined cytotoxic therapy and Rituximab because of their recognized side effects of mouth lesions, gingival bleeding, seizures and bloating. With the introduction of this treatment, the prednisolone dosage has been successfully reduced to 5 mg per day with resolution of his headaches, and control of the gingival bleeding and swelling. His MRI remains stable without changes in the white matter lesions, with no new lesions and no enhancing lesions. He has now returned to work. After 6 months of treatment the anti-MPO antibodies titres decreased to 13.6. His management is summarized in Supplementary Table 1.

## DISCUSSION

Multiple sclerosis (MS) is regarded as rare in African/Zambian people [2]. The MRI showed some lesions compatible with MS, including abnormality of the right optic nerve. At no stage did he have the clinical syndrome of optic neuritis and it was thought the optic nerve involvement was part of his vasculitic disorder. The MRI also showed subtle poorly marginated centrum semiovale white matter T2-hyperintensity, atypical for MS [3]. He also did not display any dispersion of lesions in time and space characteristic of MS.

This patient is not the representation of the possible rare association of radiologically isolated syndrome (RIS) and ANCA positive vasculitis of the mouth for the following reasons: (i) the MRI scans are not typical of MS and show no contrast enhancement; (ii) there are no cervical spinal cord lesions supportive of the propensity to MS; (iii) OGCBs were found in the CSF, indicative of active inflammation and plasma cell formation of antibodies to an antigenic stimulus, i.e. ANCA positive vasculitis and not seen in RIS; (iv) MS and probably RIS are rare in Zambians; (v) and a definite cause for cerebral vasculitis, namely ANCA+ vasculitis has been identified—these observations excluding RIS on current diagnostic criteria.

The differential diagnosis includes uncommon white matter diseases and infections (see Supplementary Tables 2 and 3). He did have OGCBs but these have been recognized in vasculitis of the brain [4]. There are no comprehensive prospective published studies on OGCBs in ANCA related vasculitis and of primary angiitis of the brain in general [5–7]. A study of 8 patients with cerebral vasculitis revealed 2 subjects with ANCA+ vasculitis, one of which had OGCBs [8]. The patient’s Zambian origin, stability of brain and mouth lesions with IVIg, lack of dispersion of white matter lesions in space and time on clinical and MRI criteria, and exclusion of all differential diagnostic infections, inflammatory and white matter diseases confirm the diagnosis of ANCA vasculitis of the brain and mouth in our patient (see Supplementary Tables 2 and 3). The MRI did not demonstrate vessel wall enhancement, vascular irregularity or haemorrhages as might be found in cerebral vasculitis, but their absence does not exclude the diagnosis.

Interestingly, the HLA-DRB1*13 haplotype has been suggested as having a protective role in MPO-ANCA+ vasculitides in Japanese patients [9]. Our Zambian patient was homozygous for DRB1*13, indicating heterogeneity in population differences in the role played by HLA-class II antigens in disease and opens up future research into the role of HLA-class II in Africans with vasculitis of the CNS.

In ANCA-related CNS inflammation, infiltration and granulomatosis may occur. The vasculitis is usually multi-system and this is supported by our patient who had involvement of the brain and oral cavity. White matter hyperintensities are recognized microvascular cerebral lesions in ANCA vasculitis of the brain [1, 10].

Here we describe for the first time an African/Zambian man with ANCA+ C-type myeloperoxidases antibody vasculitis of the brain and oral mucosa, who responded to steroids and intravenous immunoglobulin. Characteristic CNS involvement in ANCA vasculitis is usually that of hypertrophic pachymeningitis, strokes, hypophysitis, posterior reversible encephalopathy syndrome, mass lesions or myelopathy [1].

## Supplementary Material

Supplementary_tables_final_omad100

## Data Availability

The anonymised data collected are available from the corresponding author upon request.
