# Supplementary material for: Anti-neutrophil cytoplasmic antibody associated vasculitis of the brain and oral cavity: a case report
Source: Oxf Med Case Reports. 2023 Sep 25;2023(9):omad100. doi: 10.1093/omcr/omad100 (PMC10530312; doi:10.1093/omcr/omad100)
Supplement: Supplementary_tables_final_omad100 [file supplementary_tables_final_omad100.docx]

**Supplementary Table 1: Management timeline**

| **Date** | **Management** |
| --- | --- |
| April 2021 | Headaches and white matter lesions |
| June 2021 | MPO antibodies detected in high titre |
| September 2021 | Prednisolone initiated |
| October 2021 | Gingival biopsy consistent with vasculitis |
| December 2021 | C-ANCA + MPO + vasculitis brain and oral cavity diagnosed |
| January 2022 | IVIg commenced |
| Present | To this day, in remission on IVIg and prednisolone |

**Supplementary Table 2:**

**Differential diagnoses of white matter diseases of the central nervous system**

| **White Matter Diseases** | |
| --- | --- |
| - Anti-AQPO4 disease - Anti-MOG disease - Vasculitis - Anti-phospholipid antibody syndrome - ADEM - Microvascular ischaemic disease - Migraine related atypical lesions - Amyloid angiopathy - Sarcoid - SLE - Sjogren’s syndrome - B12 deficiency - N_2_O toxicity - Copper (Cu) deficiency - Behϛet’s - CADASIL/CARASIL - Susac’s disease - Toxic:   - Chronic alcohol use   - Carbon monoxide intoxication   - Toluene inhalation   - Heroin and cocaine   - Methotrexate-related leuko-encephalopathy - Metabolic:   - Acute intermittent porphyria   - Hepatic encephalopathy   - Hashimoto’s encephalopathy - Neoplastic:   - Glial tumours   - CNS lymphoma - Trauma:   - Radiotherapy   - Traumatic axonal injury | - Genetic: - Lysosomal storage diseases   - Metachromatic leukodystrophy   - Krabbe disease   - Fabry disease   - Gangliosidosis   - Mucopolysaccharidosis - Peroxisomal disorders   - X-lined adrenoleukodystrophy   - Zellweger syndrome   - Refsum disease - Mitochondrial disorders   - MERF   - MELAS   - Leigh disease   - Kearns-Sayre - Aminoacidopathies and organic acidopathies   - Canavan disease (megaloencephalopthy)   - Glutaric aciduria   - Urea cycle disorders - Alexander disease (GFAP mutations) - Van der Knapp disease (megaloencephalopathy with subcortical cysts) – mutation in MLC1. - Vanishing white matter disease (eIF2B mutations) - Myotonic dystrophy      - **Rare diseases:** - Marburg variant of MS - Balo’s concentric sclerosis - Schilder’s disease |

**Supplementary Table 3:**

**Differential diagnoses of white matter infections of the central nervous system.**

| **Infections** |
| --- |
| - HIV encephalopathy - HSV encephalitis - CMV encephalitis - Neurosyphilis - Cryptococcosis - Progressive multiple leukoencephalopathy - Whipple’s disease - Neuroborreliosis - Lyme encephalopathy - Subacute sclerosing panencephalopathy |
